# Early-life adversity accelerates cellular ageing and affects adult inflammation: Experimental evidence from the European starling

**DOI:** 10.1038/srep40794

**Published:** 2017-01-17

**Authors:** Daniel Nettle, Clare Andrews, Sophie Reichert, Tom Bedford, Claire Kolenda, Craig Parker, Carmen Martin-Ruiz, Pat Monaghan, Melissa Bateson

**Affiliations:** 1 Centre for Behaviour and Evolution & Institute of Neuroscience, Newcastle University, Newcastle, NE2 4HH, UK; 2 Institute of Biodiversity, Animal Health and Comparative Medicine, University of Glasgow, Glasgow G12 8QQ, UK; 3 Department of Animal and Plant Sciences, University of Sheffield, Sheffield, S10 2TN, UK; 4 Institute of Neuroscience, Newcastle University, Newcastle, NE2 4HH, UK

## Abstract

Early-life adversity is associated with accelerated cellular ageing during development and increased inflammation during adulthood. However, human studies can only establish correlation, not causation, and existing experimental animal approaches alter multiple components of early-life adversity simultaneously. We developed a novel hand-rearing paradigm in European starling nestlings (Sturnus vulgaris), in which we separately manipulated nutritional shortfall and begging effort for a period of 10 days. The experimental treatments accelerated erythrocyte telomere attrition and increased DNA damage measured in the juvenile period. For telomere attrition, amount of food and begging effort exerted additive effects. Only the combination of low food amount and high begging effort increased DNA damage. We then measured two markers of inflammation, high-sensitivity C-reactive protein and interleukin-6, when the birds were adults. The experimental treatments affected both inflammatory markers, though the patterns were complex and different for each marker. The effect of the experimental treatments on adult interleukin-6 was partially mediated by increased juvenile DNA damage. Our results show that both nutritional input and begging effort in the nestling period affect cellular ageing and adult inflammation in the starling. However, the pattern of effects is different for different biomarkers measured at different time points.

Experiencing adverse conditions during early life is associated with reduced survival and an increase in morbidity from many different sources, even if the adult environment is benign. For example, in humans, childhood poverty and maltreatment are associated with premature mortality and an increased risk of major non-communicable diseases such as coronary heart disease^1^,^2^,^3^. In wild non-human animals, poor conditions during early development are associated with reduced survival and lower fitness^4^,^5^,^6^. In experimental animal models, maternal separation and early weaning increase disease risk in later life^7^,^8^. The effects of early-life adversity are akin to those of ageing, since the diseases that early-life adversity makes more probable are diseases whose incidence increases with chronological age. Early-life adversity is also associated with increases in inflammation in adulthood^9^,^10^,^11^,^12^. This is related to the associations described above, since inflammation itself predicts mortality and morbidity^13^,^14^.

The impact of early-life adversity may be measurable at the cellular level long before the onset of adult disease and senescence. Specifically, studies from multiple taxa have shown that exposed individuals show accelerated telomere attrition in proliferating tissues^15^,^16^,^17^,^18^,^19^,^20^,^21^. Telomeres are the repetitive DNA caps at the ends of eukaryotic chromosomes. They shorten with age, and their attrition is accelerated by oxidative and physiological stress^22^,^23^. Telomere length or attrition prospectively predict future survival^18^,^24^,^25^. In one avian study, it was telomere length at the end of the developmental period that was the best predictor of future life expectancy^26^. Thus, several authors working from different perspectives have suggested that telomere attrition may serve as an integrative marker of the negative impact of an individual’s experience on its state, and hence of that individual’s future prospects^27^,^28^,^29^,^30^. Since telomeres shorten most rapidly during early life^26^, there is scope for exposures during this period to have a particularly large impact^31^.

Evidence from humans for links between early-life experience, telomere attrition and adult health is necessarily correlational. Thus, the possibility can never be ruled out that the individuals experiencing early-life adversity are a non-random component of the population, and thus that associations with early adversity are non-causal by-products of common genetic or environmental third factors^3^. In altricial birds, it is possible to manipulate early-life conditions experimentally, and this provides stronger evidence for the causal impact of adversity on telomeres. Several experimental studies have manipulated brood size^18^,^19^,^32^. Enlarging the brood is likely to have multiple effects, including reducing the per capita food supply^19^,^33^,^34^ as well as increasing the level of begging^35^. Thus, these studies do not allow us to resolve what exactly it is about large broods that has the negative impact on telomeres. In one of our previous experiments, we manipulated the position of the focal nestling in the brood hierarchy (i.e. whether it was larger or smaller than its competitors)^20^. We found that being smaller than competitors accelerated telomere attrition but did not affect weight gain. Given that smaller nestlings have to beg more to elicit parental investment, this result suggests that negative effects of nestling experience are not solely due to the amount of nutritional input obtained, but are also affected by the begging the nestling has to perform. The present experiment was designed to examine the influences of nutritional inadequacy and begging effort on nestling development in a more controlled way, by varying them independently.

We hand-reared nestlings from days 5 to 15 post-hatching, independently varying two types of adverse experience, the amount of food provided (henceforth, *Amount;* either *Plenty* or *Lean*), and the begging effort required to obtain it (*Effort*; *Easy* or *Hard*). *Amount* was manipulated by restricting nestlings in the Lean groups to approximately 73% of the food given to the Plenty groups, who were fed to satiety, on their most recent feed^36^. *Effort* was manipulated by stimulating approximately twice the daily duration of begging from the Hard groups as from the Easy groups. Amount and Effort were combined factorially to give four experimental groups: Lean-Hard, Lean-Easy, Plenty-Hard, and Plenty-Easy. Hence, our experimental treatments involved types of adverse experience that occur naturally in our study species, but allowed the causal separation of factors that are usually confounded in the wild and in brood-size manipulation experiments. We measured the attrition of erythrocyte telomeres over the course of the manipulation, and in the juvenile period immediately following it. We also simultaneously measured a marker of oxidative damage to DNA, 8-hydroxy-2′-deoxyguanosine (8-OHdG) at the same time points. The motivation for measuring oxidative damage to DNA is that an increase in such damage is one of the pathways through which organism-level stress may accelerate the shortening of telomeres^28^. The juvenile measurements of telomeres and oxidative damage were intended to capture the possibility there might be ongoing impacts after the manipulation had ended, for example due to catch-up growth or other compensatory processes^37^. Once the birds were adults, we measured inflammation via two markers, high-sensitivity C-reactive protein (HS-CRP), and interleukin-6 (IL-6). Our main research questions were: first, do either Amount, Work or their combination affect telomere attrition and DNA damage during and immediately after the experimental manipulation; second, do either Amount, Work or their combination affect inflammation in adulthood; and third, are any effects of Amount and Work on inflammation in adulthood statistically mediated by telomere attrition or DNA damage in the juvenile period?

## Results

### Weight gain and skeletal growth

**Figure 1:**
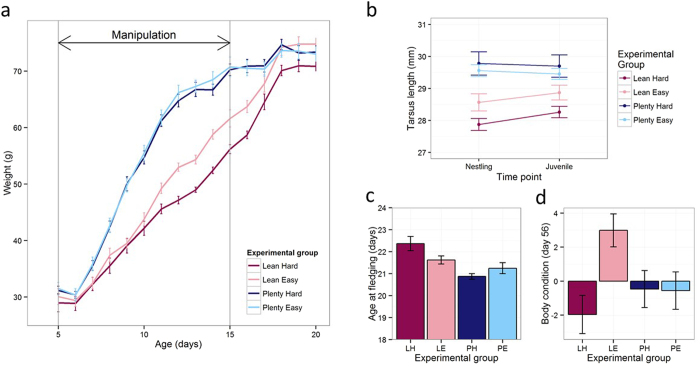
Effects of experimental treatments on development. Error bars represent one standard error. (**a**) Mean daily weight by experimental group during and immediately after the manipulation. (**b**) Mean tarsus length by experimental group as nestlings (day 15) and juveniles (day 56). (**c**) Mean age at fledging in days by experimental group. (**d**) Body condition (residual of weight from skeletal size) by experimental group as juveniles (day 56).

**Figure 2:**
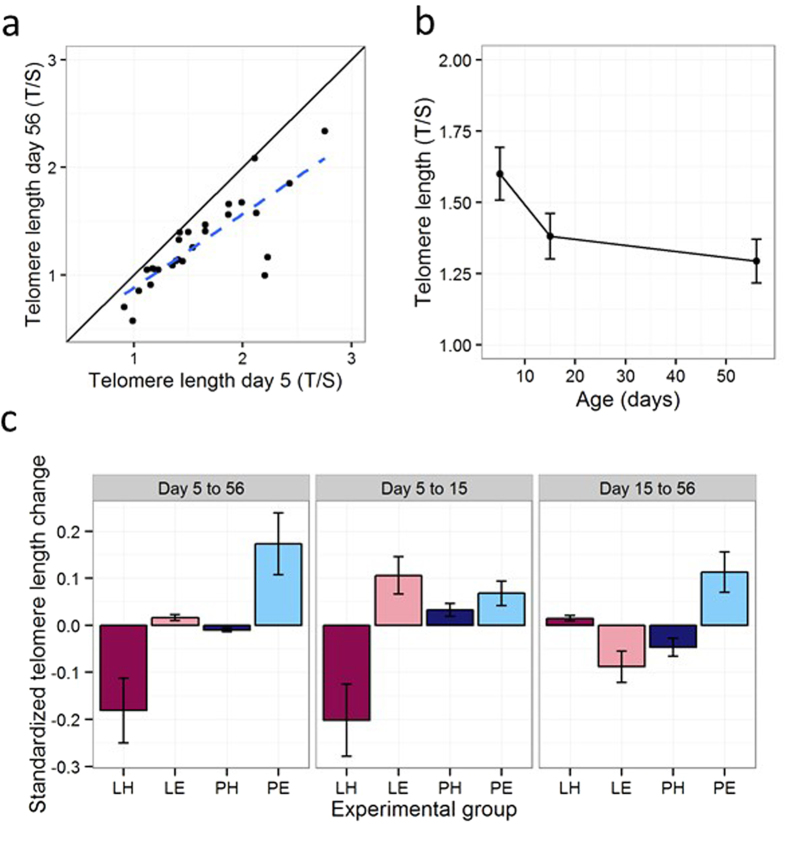
Telomere dynamics. (**a**) Relationship between juvenile telomere length (day 56) and telomere length at day 5. The solid line represents x = y. If there were no overall attrition, observations would be expected to fall around this line. The dashed line represents the least-squares line through the data. (**b**) Mean telomere lengths at days 5, 15 and 56. Error bars represent one between-bird standard error. (**c**) Standardized change in telomere length (D) by experimental group over the whole developmental period (days 5 to 56), the period of the experimental manipulation (days 5 to 15), and the post-manipulation period (days 15 to 56). Error bars represent one standard error.

Both experimental treatments affected weight over the course of the manipulation (Fig. 1a). The Lean groups gained weight more slowly than the Plenty groups, the Hard groups gained weight more slowly than the Easy groups, and the Lean-Hard group gained weight particularly slowly (Supplementary Information, Table S1; Amount*Age interaction: likelihood ratio test [LRT] = 125.58, P < 0.01; Effort*Age interaction: LRT = 8.46, P < 0.01; Amount*Effort*Age interaction: LRT = 7.04, P < 0.01). Skeletal size at day 15 as assessed by tarsus length was affected by Amount (Table S2; LRT = 24.79, P < 0.01) but not Effort, with Lean birds smaller than Plenty (Fig. 2b). Although Lean birds showed some post-day-15 catch-up growth not seen in Plenty birds, they remained skeletally smaller than Plenty birds at day 56 (Fig. 1b; Table S2; LRT = 18.33, P < 0.01). Fledging was delayed in the Lean birds compared to Plenty, and the Lean-Hard group were significantly more delayed than the Lean-Easy (Fig. 1c; Table S3; Amount: LRT = 9.92, P < 0.01; Amount*Effort interaction: LRT = 6.69, P < 0.01). By day 56, the Lean-Easy birds had more than compensated for their nutritional restriction, and were significantly heavier than the other groups, both in absolute terms and after controlling for their skeletal size, whilst the Lean-Hard group remained relatively light for their skeletal size (Fig. 1d; Table S4; Amount*Effort interaction: LRT = 7.52, P < 0.01).

### Telomere attrition

Telomere lengths on day 56 were strongly correlated with telomere lengths on day 5 (r_25_ = 0.82, P < 0.01; Fig. 2a). There was nonetheless attrition over the course of development, with telomeres shorter on day 56 than at day 5 (paired t-test: t_26_ = −5.76, P < 0.01; Fig. 2a). Figure 2b shows that the bulk of the attrition occurred between day 5 and day 15: on average, T/S ratios changed 2.53 as much in the 10 days from day 5 to day 15 as in the 41 days from day 15 to day 56. There were significant main effects of both Amount (Table S5; Lean Amount associated with greater telomere loss than Plenty; LRT = 7.14, P < 0.01) and Effort (Hard Effort associated with greater telomere loss than Easy; LRT = 5.20, P = 0.02) on telomere length change from day 5 to day 56. There was no significant interaction between Amount and Effort. Thus, the group whose telomeres shortened the most overall was Lean-Hard, and the group with the least telomere attrition was Plenty-Easy, with the other two groups intermediate (Fig. 2c). Individuals with longer telomeres also experienced more shortening (Table S5; LRT for day 5 telomere length as a predictor of shortening = 6.85, P < 0.01).

We also separately analysed telomere length change during the experimental manipulation itself (day 5 to day 15), and in the period after the manipulation (day 15 to day 56; Fig. 2c). During the manipulation, Hard Effort significantly increased telomere loss (LRT = 3.84, P = 0.048), the effect of Amount was marginally nonsignificant (LRT = 3.48, P = 0.06), and there was no interaction (Table S5). Post-manipulation, telomere length change continued to be affected by the interaction of Amount and Effort (Amount*Effort interaction, LRT = 13.12, P < 0.01; Table S5): among the Plenty birds, the Plenty-Hard group experienced more loss than the Plenty-Easy group, whereas Among the Lean birds, the Lean-Easy group showed greater loss than the Lean-Hard group. Variation in the rate of telomere attrition between days 15 and 56 was apparently not explained by the extent of post-manipulation mass gain; when the difference between day 56 and day 15 mass was added to the model, its effect was not significant (LRT = 0.13, P = 0.72), and the Amount*Effort interaction remained significant (LRT = 12.65, P < 0.01).

### Oxidative damage

**Figure 3:**
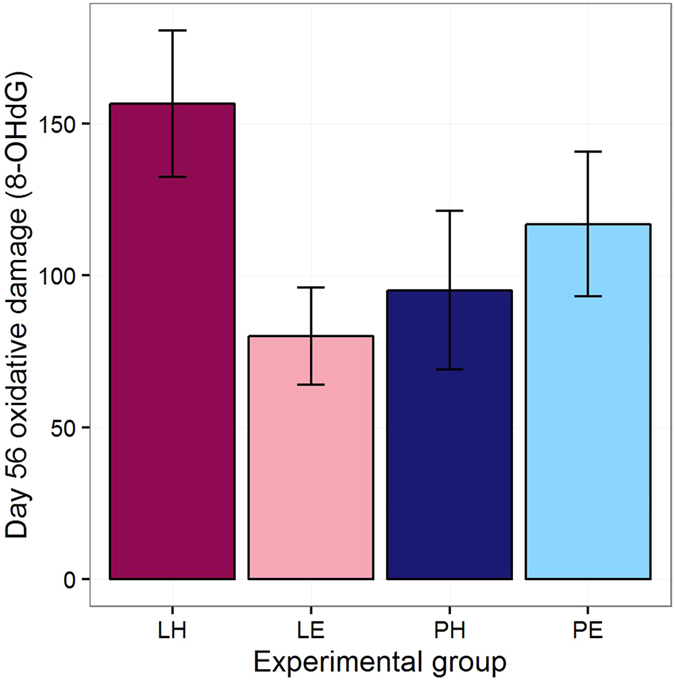
Oxidative damage to DNA (pg 8-OHdG/μg DNA) at day 56, by experimental group. Error bars represent one standard error.

We assessed oxidative damage to DNA by measuring 8-OHdG on days 5, 15 and 56. The 8-OHdG measures at the three time points were positively correlated with one another, significantly in two cases and non-significantly in the other (day 5 to day 15, r = 0.24, P = 0.21; day 15 to day 56, r = 0.52, P < 0.01; day 5 to day 56, r = 0.59, P < 0.01). None of the correlations between the 8-OHdG measures and the telomere length change measures was significant (r values between −0.07 and 0.36, P ≥ 0.07). There were no significant treatment effects on 8-OHdG on days 5 or 15 (Table S6). However, on day 56, there was a significant interaction between Amount and Effort (LRT = 11.40, P < 0.01), with the Lean Hard birds showing greater levels of 8-OHdG than all other experimental groups (Fig. 3).

### Inflammation in adulthood

**Figure 4:**
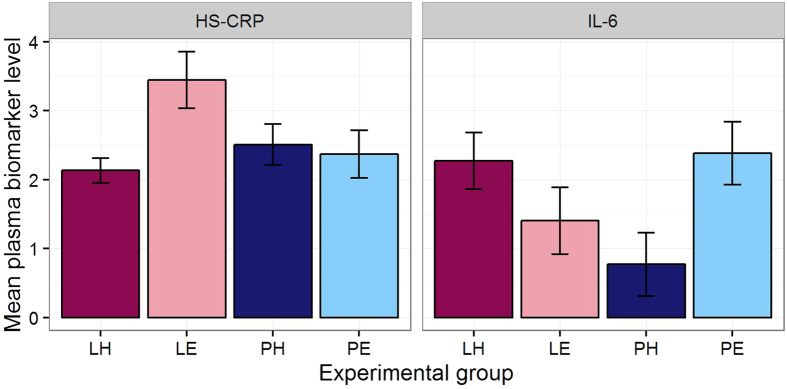
Inflammation markers in adulthood. Mean high sensitivity C-reactive protein (HS-CRP; square-root μg/ml) and interleukin-6 (IL-6, log pg/ml) in adult plasma, by experimental group. Error bars represent one standard error.

**Figure 5:**
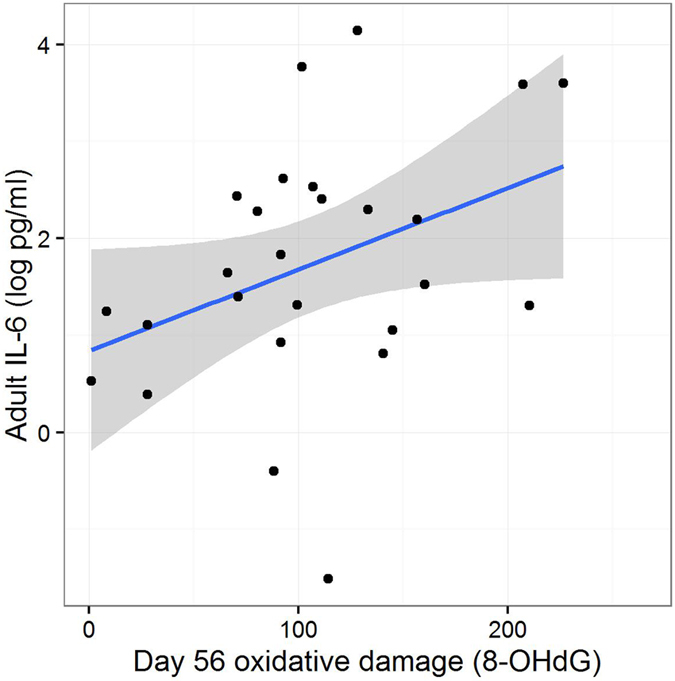
Adult plasma interleukin-6 (IL-6, log pg/ml) against day 56 oxidative DNA damage (pg 8-OHdG/μg DNA). The line represents a least-squares regression line and the shaded area its 95% confidence interval.

The two markers of adult inflammation, HS-CRP and IL-6, were not significantly correlated with one another (r = −0.14, P = 0.51). There was a significant interaction between Amount and Effort in predicting both markers (Table S7; HS-CRP: Amount*Effort interaction, LRT = 5.05, P = 0.02; IL-6: Amount*Effort interaction, LRT = 8.33, P < 0.01). The interactions were somewhat different in the two cases (Fig. 4). For HS-CRP, Lean Amount was associated with higher inflammation only when coupled with Easy Effort; the Lean-Hard group appear to have been protected. For IL-6, the Lean-Hard group showed higher levels than the Lean-Easy group, and unexpectedly, in the Plenty Treatment, the Plenty-Easy group showed higher levels than the Plenty-Hard group. We explored whether adult inflammation was predicted by telomere length change (using the day 5 to day 56 measure) and 8-OHdG (using the day 56 measure) during development (Table S8). Telomere length change did not significantly predict either marker of adult inflammation, and day 56 8-OHdG did not significantly predict HS-CRP. However, day 56 8-OHdG did significantly predict adult IL-6 (LRT = 6.95, P < 0.01), with higher day 56 8-OHdG associated with greater HS-CRP in adulthood (Fig. 5). When day 56 8-OHdG was entered into the same statistical model for IL-6 with the developmental treatments, the parameter estimate for the interaction between Amount and Effort was attenuated from −2.40 (s.e. 0.71) to −1.82 (s.e. 0.74). This represented significant partial mediation (Sobel test = −2.07, P = 0.04). The interaction between Amount and Effort nonetheless remained significant (LRT = 5.42, P = 0.02).

## Discussion

Our experimental paradigm allowed us to separate the effects of different types of early-life adverse experience, namely nutritional inadequacy (Lean Amount) and high begging effort (Hard Effort), on development and cellular ageing. Only Lean Amount, not Hard Effort, affected final skeletal size, suggesting that the Effort treatments had not substantively impaired energy availability for growth. (The Hard Effort groups gained weight more slowly than the Easy ones during the manipulation, which reflects the energetic cost of begging^38^, but the lack of effect on skeletal size suggests this was not sufficient to limit structural growth.) Both Amount and Effort affected telomere attrition and oxidative damage to DNA over the course of development. The effects were not restricted to the period of the manipulation itself, but extended into the post-manipulation period (indeed, were restricted to the post-manipulation period for DNA damage), suggesting that the costs of early-adversity may to a considerable extent be attributable to subsequent knock-on or compensatory processes. We also found evidence that both experimental treatments affected inflammatory markers measured when the birds were adults. For IL-6, this effect was partially mediated by the increase in DNA damage during development. Overall, in answer to the question of what it is about large broods that has a negative impact on nestlings in altricial bird species, the reduction in the food supply or the additional begging, we can tentatively answer: both. More generally, our results confirm the causal importance of early-life exposures for cellular ageing during development, and inflammation in adulthood. Significantly, our manipulations were naturalistic; all groups grew at rates that are within the observed variation in un-manipulated wild nests^39^, and large nest-to-nest variation in the amount of begging is very apparent in the field (personal observations). Thus, we have some confidence that our treatments fall within the range of frequent natural experience for the species, and hence that the consequences we found are relevant to variation in life histories in natural populations.

The effects of Lean Amount and Hard Effort on telomere attrition overall were additive, so that the group experiencing both adversities showed more attrition than the two groups with one adversity but not the other, who showed more than the group with neither. The timing of the accelerated attrition depended on the combination of Amount and Effort: Lean Amount coupled with Hard Effort produced an acceleration already detectable by day 15, whereas Lean Amount coupled with Easy Effort or Plenty Amount coupled with Hard Effort produced acceleration during the post-manipulation period (days 15 to 56). The extent of variation in post-manipulation telomere attrition did not appear to be explained by the extent of post-manipulation weight gain. We confirmed previous observations that longer telomeres shorten faster, even when regression to the mean has been corrected for^40^, and that the bulk of attrition happens very early^26^; in our case, much more occurring during the period of linear growth (days 5–15) than between days 15 and 56. This accords with the assumptions of the High Initial Damage Load hypothesis, which states that very early life is a period when the background rate of somatic ageing is high, and therefore that sensitivity to any factors affecting that rate is very high during the early-life period^31^.

For DNA damage, there was no evidence of an increase due to experimental treatment during the manipulation itself, but the consequences of the treatments became manifest in the period after the manipulation had ended. Only the combination of Hard Effort and Lean Amount increased DNA damage; in isolation, neither adversity type showed significant evidence of impact. Our measures of DNA damage did not significantly predict the rate of telomere attrition. We had expected they might, since oxidative damage to DNA is one of the processes affecting the rate at which telomeres are eroded^28^. This may suggest that other pathways—such as the direct effect of stress hormones on production of the telomere repair enzyme telomerase^41^—may be important in explaining the accelerated attrition we observed in the Hard and Lean treatments.

We measured inflammation in adulthood using two plasma markers, HS-CRP and IL-6. Though both are widely used as indicators of chronic inflammation, IL-6 is considered the less general marker of overall inflammatory activity, and its associations with subsequent morbidity tend to be weaker^42^,^43^. The two markers were not strongly correlated with one another. The interaction between Amount and Effort affected both markers, and to this extent our results accord with the widespread finding that early developmental factors are associated with adult inflammatory phenotype^3^,^10^,^44^. However, the pattern of results did not conform to our predictions, was different across the two markers, and is not straightforward to interpret. It was not that the two types of adversity in combination exerted an effect greater than either in isolation. Instead, for HS-CRP, it was the Lean-Easy group that showed the highest levels, whilst the Lean-Hard were not different from the two Plenty groups. This may relate to the relatively high juvenile body weights of this group (discussed below), since C-reactive protein levels are strongly associated with adiposity^45^. For IL-6, the Lean-Hard group showed high levels, but so, unexpectedly, did the Plenty-Easy group. Average inflammatory activity at any point in adult life is likely to be shaped by a number of factors to do with behaviour, activity, diet, and body composition. Thus, the unpredicted pattern of results may represent early-life experience affecting adult behavioural or physiological parameters that in turn have consequences for inflammatory functioning, as well as, or instead of, greater early-life adversity leading directly to a more pro-inflammatory phenotype.

The complexity of the relationship between early adversity and adult inflammation is further highlighted by the fact that, although juvenile DNA damage levels predicted adult IL-6, developmental telomere attrition did not predict any aspect of adult inflammation. We predicted that it would do so, since developmental telomere attrition has been proposed as an integrative marker of the impact of adversity during development^27^,^28^,^29^,^30^, a proposal supported by our results on the effects of the experimental treatments on telomere attrition. Thus, we expected developmental telomere attrition to be at least as strong a predictor of adult inflammation as the experimental treatments themselves. The null nature of the associations is partly an issue of statistical power: the correlations between developmental telomere attrition and the adult inflammation markers were in the predicted direction, albeit far from statistical significance with the present sample size. Nonetheless, the fact that the associations appear to be so weak is surprising given that both developmental telomere attrition and chronic inflammation are biomarkers of ageing^46^, and have been found to predict future morbidity and mortality^14^,^25^,^26^.

Our results produced the incidental observation that the Lean-Easy group were heavy for their size as juveniles. The developmental induction of high adult body weight by early-life adversity has been observed in many species, including humans^47^,^48^,^49^,^50^,^51^, and our own previous work in starlings^52^. Our results here showed that induction of relatively high body in the juvenile starling requires not just early nutritional restriction, but the combination of early nutritional restriction and a low level of effort required to obtain food. Early nutritional restriction coupled with high effort produces the opposite result, juvenile birds that are light for their skeletal size. We are currently studying how long these weight differences persist through adulthood.

Our experimental paradigm had a number of limitations. All nestlings experienced early parental separation, and therefore no group represents an un-manipulated control. However, altricial passerines can be very readily hand-reared if taken prior to filial imprinting, and patterns of growth and development were in line with our previous studies of parent-reared nestlings^19^,^20^,^39^. Moreover, hand-rearing cannot account for the differences amongst experimental groups. A second limitation is that the Hard Effort groups received more disturbance in general, as well as performing more begging. This was unavoidable, since the nestlings could not be made to beg without disturbing them, and could not be disturbed in any way that did not trigger begging. Thus, there was no way to separate the consequences of begging from those of disturbance in general. However, since the nestlings had imprinted on the researchers, there is no reason to believe that our nest visits were intrinsically stressful to them. A more major limitation is that we have not shown how our markers of cellular ageing, or indeed adult inflammation, relate to actual longevity in this cohort. However, telomere attrition or length has been shown to predict longevity in several avian^18^,^25^,^26^ and non-avian systems^24^. Thus, there are reasonable grounds for believing that the variation we have documented in cellular ageing and inflammation would be associated with subsequent mortality and morbidity.

Although we found evidence that both nutritional inadequacy and begging effort contribute to the effects of early-life adversity on cellular ageing and adult inflammation, we found several cases where their effects combined non-additively. Moreover, the patterns of effects of the two treatments were rather different across telomere attrition, DNA damage, and the two markers of adult inflammation. In observational studies of early-life adversity and ageing, different types of adversity are often summed together into a single adversity score^11^,^53^,^54^; and different biomarker outcomes are often collapsed to make a single measure of biological age^46^,^55^. Whilst this may be convenient for detecting broad relationships, our results suggest that each biomarker outcome may be affected by specific components of early adversity in a slightly different way; and that the effect of a combination of types of early adversity may often be different from the sum of the effects of the two types considered in isolation. Using summary measures of adversity to predict summary measures of health or biological age would not adequately capture these complexities.

## Methods

### Subjects

Subjects were 32 European starlings, *Sturnus vulgaris*, taken from 8 wild nests in monitored nesting boxes in Northumberland under Natural England permit 20121066. The study was approved by the Animal Welfare and Ethics Board at Newcastle University and the UK Home Office (licence PPL 70/8089). The study was carried out in accordance with the Home Office licence and the Association for the Study of Animal Behaviour (ASAB) guidelines for the use of animals in research. All birds fledged successfully, but two (1 × Lean-Easy, 1 × Lean-Hard) died prior to 20 months. The European starling is a gregarious, medium-sized passerine with a maximum longevity of 22.9 years given in the AnAge database^56^. The four nestlings from each nest were likely to have been full siblings, since extra-pair fertilizations and intra-specific brood parasitism are relatively rare^57^. Nestlings were collected on day 5 and one member of each family was assigned at random to 4 experimental groups of 8 birds (Plenty-Easy, Plenty-Hard, Lean-Easy, Lean-Hard) on arrival. Each experimental group occupied two separate covered artificial nests of 4 nestlings. Arrival weight did not differ by experimental treatment (linear mixed model: Amount: LRT = 2.63, P = 0.11; Effort: LRT = 2.67, P = 0.11; Amount*Effort: LRT = 0.67, P = 0.41). Nestlings were uniquely marked, initially with non-toxic correcting fluid, then with plastic leg rings applied on day 7.

### Experimental manipulation

From the day after arrival, experimental groups received nine feeds a day. In the Plenty groups, each feed was to satiety, whilst for the Lean groups the amount fed was approximately 73% that fed to the corresponding Plenty group on the most recent feed. The Hard groups additionally received nine ‘sham’ feeds each day. During these sham feeds, which had approximately the same duration as a genuine feed (2 minutes), nestlings were stimulated to beg, but no food was delivered.

#### Feeds

At regular intervals between approximately 07:00 and 21:00 each day, the nest lid was removed and all nestlings delivered standardized 0.5 ml aliquots of food via a 25 ml Eppendorf repeater pipette. Food consisted of a blended mixture of high-meat cat food, apple sauce and vitamins, a mixture previously shown to be appropriate for hand-rearing starlings^58^. For the Plenty groups, more aliquots were delivered until the nestling refused further feeding, and the number per individual noted. The number of aliquots for each individual in the Lean groups was calculated as a proportion of the mean consumption of the corresponding Plenty group on the same feed. Initially the proportion was set to 70%^36^, but this was dynamically adjusted each day so that the weight gain of the Lean birds tracked that of the lightest nestlings in a previous study of wild-reared nestlings^19^. The total amount fed to the Lean groups over the whole manipulation was 72.75% that of the Plenty groups.

#### Sham feeds

For the Hard groups, at nine points each day evenly interspersed between the feeds, we removed the nest lid and used the feeding pipette to stimulate the nestlings to beg for two minutes (the approximate duration of a feed) without delivering any food. All nestlings begged readily for at least part of the time.

#### Post-manipulation

The experimental manipulations were continued until day 15 (after 7^th^ feed), when nestlings were regrouped by natal family rather than experimental group. From day 15 until fledging (around day 21), all nests were fed to satiety on a constant rotation. Once birds had fledged, they were kept in mixed-treatment cages until they had been observed eating for themselves, and then moved to permanent large mixed-treatment aviaries (215 × 340 × 220 cm; ~18 °C; 40% humidity; 15 L: 9D light cycle) in flocks of 16.

#### Biometric data and blood sampling

During the experimental manipulation, nestlings were weighed prior to the first feed each morning using on a digital balance. Juveniles were caught and weighed in the same manner on day 56. Skeletal size was assessed at day 15 and again at day 56 by measuring tarsi using digital calipers, with two independent measurements of each tarsus on each occasion. The final variable for each time point represents the average of the four measurements. Blood samples (70–120 μl) were taken by puncture of the alar or metatarsal vein on days 5, 15, 56, and again as young adults at 20 months, using a 25-gauge needle and capillary tube (heparinized at days 5, 15 and 56, EDTA-coated at 20 months). Blood samples were immediately centrifuged to separate cells from plasma, and frozen to −80 °C for later analysis.

### Outcome measures

#### Erythrocyte telomere length

Relative erythrocyte telomere length was assessed from DNA extracted from day 5, 15 and 56 blood samples using the quantitative real-time PCR amplification approach^59^ as adapted for birds^60^. In this approach, the abundance of the telomeric sequence is expressed relative to that of a known single-copy gene (in this case, GADPH), producing a single number (the T/S ratio) to express relative mean telomere length in each sample. Samples were assayed in triplicate and the mean used. Serial dilutions of a pooled DNA standard were included on each of 7 plates to generate a reference curve to control for amplifying efficiency. Mean amplification efficiency calculated from the reference curves of the qPCR runs were between 107–112 (telomere) and 106–115 (control gene). R^2^ calculated from the reference curves were 0.98–0.99 both for the telomere and the control gene assays. Intra-plate mean coefficients of variation (CVs) for Ct values were 1.7% (telomere assay) and 0.4% (control gene assay). Inter-plate CVs for Ct values based on repeated samples were 2.7% (telomere assay) and 0.7% for (control gene assay). T/S ratios were calculated using the ΔΔCt method^59^. Calculation using the approach of Pfaffl^61^, which incorporates variation in amplification efficiency, produced virtually identical results (*r* > 0.99). Plate accounted for no variation in T/S ratios once effects of individual and natal family were accounted for. Mean CV for the relative T/S ratios was 18%. For 5 birds (1 or 2 from each experimental group), the GAPDH assay failed and they were removed from the analysis. We have encountered this issue in previous studies of the same population^19^,^20^,^39^, and it may represent mutations to the GAPDH gene inhibiting its amplification with the current primer.

#### Oxidative damage to DNA

We determined oxidative damage to DNA from day 5, 15 and 56 samples by measuring 8-hydroxy-2′-deoxyguanosine (8-OHdG), using the colorimetric assay EpiQuik™ 8-OHdG DNA Damage Quantification Direct Kit (EpiGentek Group Inc. Farmingdale, NY USA). When the DNA base guanine is damaged by oxidation, it is excised by repair mechanisms; excised bases from all tissues go into the circulation before being excreted. The assay measures plasma abundance of these excised oxidized bases. Whilst there is some debate on the interpretation of the assay^62^, it is generally taken as a measure of the extent of oxidative damage to DNA^63^. DNA damage measurements are expressed in pg/μg of DNA. The mean intra-plate CV was of 4.6%.

#### Inflammation in adulthood

Plasma samples recovered from EDTA blood taken at age 20 months were assessed for the two inflammation markers HS-CRP and IL-6 by solid phase sandwich enzyme-linked immunosorbent assay (ELISA) protocols, using a pigeon HS-CRP ELISA Kit (MyBiosource, San Diego, CA, USA), and a chicken IL-6 ELISA Kit (MyBiosource, San Diego, CA, USA). Both are widely used as plasma markers of chronic inflammation: IL-6 is an inflammatory cytokine, whilst CRP is an acute phase protein involved in the inflammatory response. HS-CRP concentration was expressed in μg/ml and IL-6 in pg/ml. The respective inter-assay CVs were 3.1% and 8.3%. Two birds had died by 20 months, and for a further four samples (2 × Lean-Easy, 1 × Plenty-Easy, 1 × Plenty-Hard), plasma volume was insufficient to obtain an IL-6 value.

### Molecular sexing

Sex of starling nestlings is not phenotypically obvious and so experimental groups could not be balanced for sex. Instead, birds were sexed genetically to control statistically for sex differences. Molecular sexing followed the standard approach^64^ by amplification of the chromodomain-helicase-DNA binding (CHD) genes in 20 μl real-time qPCR reactions.

### Statistical analysis

For a single-number summary of telomere shortening between sampling points, we used the D measure suggested by Verhulst *et al*.^40^. This standardized measure of change corrects for regression to the mean (zero indicates the individual experienced the average amount of change in the sample, a negative number indicates more extreme loss than average, a positive number indicates less extreme loss than average). Each of the three D values (day 5 to day 56, day 5 to day 15, day 15 to day 56) was very highly correlated with the simple difference in telomere length between the same two time points (r > 0.94). Values for both markers of inflammation were right-skewed. To improve distributions, HS-CRP was square-root transformed and IL-6 log-transformed for analysis.

Data were analysed using linear mixed models using package lme4^65^ in the R programming language^66^, with parameter estimation by maximum likelihood and inference by likelihood ratio test (LRT). Model residuals were examined for appropriate distributions. The default fixed effect structure was Sex, Amount, Effort and the Amount by Effort interaction. Departures from this default structure were as specified in the full model tables given in Supplementary Information. Random intercepts were included for natal family in all cases. For weight over the course of the manipulation, which involved repeated measurements of the same individual, there were random intercepts for individual within natal family, and fixed effects as specified in Table S1 (Supplementary Information).

## Additional Information

**How to cite this article**: Nettle, D. *et al*. Early-life adversity accelerates cellular ageing and affects adult inflammation: Experimental evidence from the European starling. *Sci. Rep.* **7**, 40794; doi: 10.1038/srep40794 (2017).

**Publisher's note:** Springer Nature remains neutral with regard to jurisdictional claims in published maps and institutional affiliations.

## Supplementary Material

Supplementary Information

Supplementary Dataset 1

Supplementary Dataset 2

Supplementary Dataset 3
